# Efficacy and sex-specific outcomes after six episodes of percutaneous tibial nerve stimulation treatment on overactive bladder syndrome symptoms

**DOI:** 10.3389/fruro.2024.1352701

**Published:** 2024-02-19

**Authors:** Carlos Perez−Martinez, Jose Luis Palacios-Galicia, Irma B. Vargas-Diaz, Alvaro Munoz, Yolanda Cruz

**Affiliations:** ^1^ Doctorado en Ciencias Biológicas, Universidad Autónoma de Tlaxcala, Tlaxcala, Tlaxcala, Mexico; ^2^ Centro de Urologia Avanzada (CURA), Delicias, Chihuahua, Mexico; ^3^ Facultad de Ciencias para el Desarrollo Humano, Universidad Autónoma de Tlaxcala, Tlaxcala, Tlaxcala, Mexico; ^4^ Centro Universitario del Norte, Universidad de Guadalajara, Colotlán, Jalisco, Mexico; ^5^ Centro Tlaxcala de Biología de la Conducta, Universidad Autónoma de Tlaxcala, Tlaxcala, Tlaxcala, Mexico

**Keywords:** urgency, urinary incontinence, neuromodulation, nocturia, OABq-SF

## Abstract

**Objective:**

This study aimed to investigate the efficacy of six episodes of percutaneous tibial nerve stimulation (PTNS) treatment for overactive bladder (OAB) symptoms in men and women and to determine the duration of the effects over a 6-month period.

**Methods:**

Women and men with OAB symptoms participated in the study, which was conducted in accordance with an approved ethical protocol (ISRCTN15733799). Patients underwent six 30-min PTNS sessions, twice per week. The symptoms of OAB were assessed using a voiding diary, the short form of the Overactive Bladder Symptom Score questionnaire (OAB-q SF), and the Patient Perception of Bladder Condition (PPBC), which were self-completed by patients before and after treatment (24 h and at 1, 3, and 6 months). The outcome data were compared for sex and time points using two-way ANOVA for repeated measures.

**Results:**

PTNS treatment significantly improved the OAB symptoms and quality of life in men and women. PTNS increased the voided volume (*p* < 0.05) and decreased the frequency of voiding, nocturia, and urgency at 24 h and up to 6 months (*p* < 0.001). The OABq-SF and PPBC scores were significantly decreased after PTNS (*p* < 0.001). Urgency was greater in men than in women. The duration of PTNS clinical success on frequency and the perception of improvement in quality of life were greater in women than in men.

**Conclusion:**

The clinical effects of six sessions of PTNS strongly suggest its potential for shortening, from the standard 12 sessions, to effectively alleviate symptoms of OAB. This therapeutic procedure would reduce both the time and economic costs of OAB treatment.

## 1 Introduction

Overactive bladder (OAB) is defined as urinary urgency, usually accompanied by increased daytime frequency and/or nocturia, with (OAB-wet) or without (OAB-dry) urinary incontinence, in the absence of urinary tract infections or other detectable diseases (1). It is a significant health problem that affects the regular daily activities of those who suffer from it, further affecting their quality of life. Moreover, depending on the severity, it may be associated with urinary tract infections, falls, and an increased number of visits to the doctor, resulting in health-related increased expenditure. Worldwide, OAB expenses have been calculated at more than 1 billion euros per year (2).

Overall, OAB is prevalent in both men and women; however, sex-specific differences exist in relation to individual OAB symptoms and in the degree of discomfort and quality of life (3). Variations in the baseline severity of OAB symptoms based on sex might affect the efficacy of treatments. However, the identification of sex-specific differences in treatment outcomes remains insufficient.

Behavioral and pharmacological treatments are the first two lines of therapy for OAB symptoms (4). The third line includes intradetrusor onabotulinum toxin A (BTX-A) injections and neuromodulation, such as sacral electrical stimulation or percutaneous tibial nerve stimulation (PTNS) (4, 5).

PTNS is a minimally invasive method that has gained interest as an alternative therapy for OAB symptoms due to its reproducibility and the low occurrence of adverse events (5). In most studies, treatments consist of 10–12 sessions, with one to three sessions per week and 30 min of electrostimulation per session. The reported efficacy varies from 37.3% to 82% (6). The positive effects of PTNS include reductions in OAB frequency, nocturia, urgency, voided volume, and urge incontinence (6, 7). However, despite several functional and clinical studies demonstrating the efficacy of PTNS, there is still no scientific or clinical support for the use of 10–12 sessions of PTNS.

It has been shown that one PTNS session can induce immediate changes in cortical activity, with its effect lasting up to 24 h (8). This neurophysiological effect suggests that 10–12 PTNS sessions may not be necessary to obtain clinical results. We hypothesize that a shortened PTNS protocol will have clinical efficacy. A reduction in the number of PTNS sessions decreases the economic costs and clinical time for OAB treatment, benefitting both patients and medical personnel.

Another important point to investigate is whether the effectiveness of PTNS in OAB treatment is sex-dependent. As mentioned above, OAB is prevalent in both men and women, but sex-specific differences exist in relation to the individual OAB symptoms. However, only a few studies included both men and women, and sex was not included as a variable for analysis (7).

The aims of the present study were to investigate the efficacy of a shortened protocol (half of the standard one) of PTNS treatment on OAB symptoms in men and women and to determine the duration of positive effects along a 6-month time frame.

## 2 Methods

### 2.1 Patient inclusion and study design

The experimental protocol was approved by the institutional Ethics Committee (protocol public registry no. ISRCTN15733799) and conformed to the provisions of the Declaration of Helsinki. All participants signed informed consent.

From January 2015 to December 2017, volunteers were invited to participate in the present study. The inclusion criteria were as follows: adults over 18 years of age with non-obstructive OAB symptoms (physical examination; uroflowmetry, ≥15 ml/s) regardless of prior anticholinergic use, but not responders to medications; with at least 8 voids over 24 h recorded using a voiding diary; and medical history. The exclusion criteria were: OAB pharmacotherapy within the last 2 weeks, good response to prior pharmacotherapy, positive urinalysis for infection, pregnancy, history of bladder cancer, bladder stones, pelvic radiotherapy, and congenital or acquired lesion of the nervous system.

A total of 125 patients responded to the invitation, and 67 of them were assessed at the urology clinic for eligibility. Only 27 volunteers met the inclusion criteria and were assigned to PTNS.

**Figure 1:**
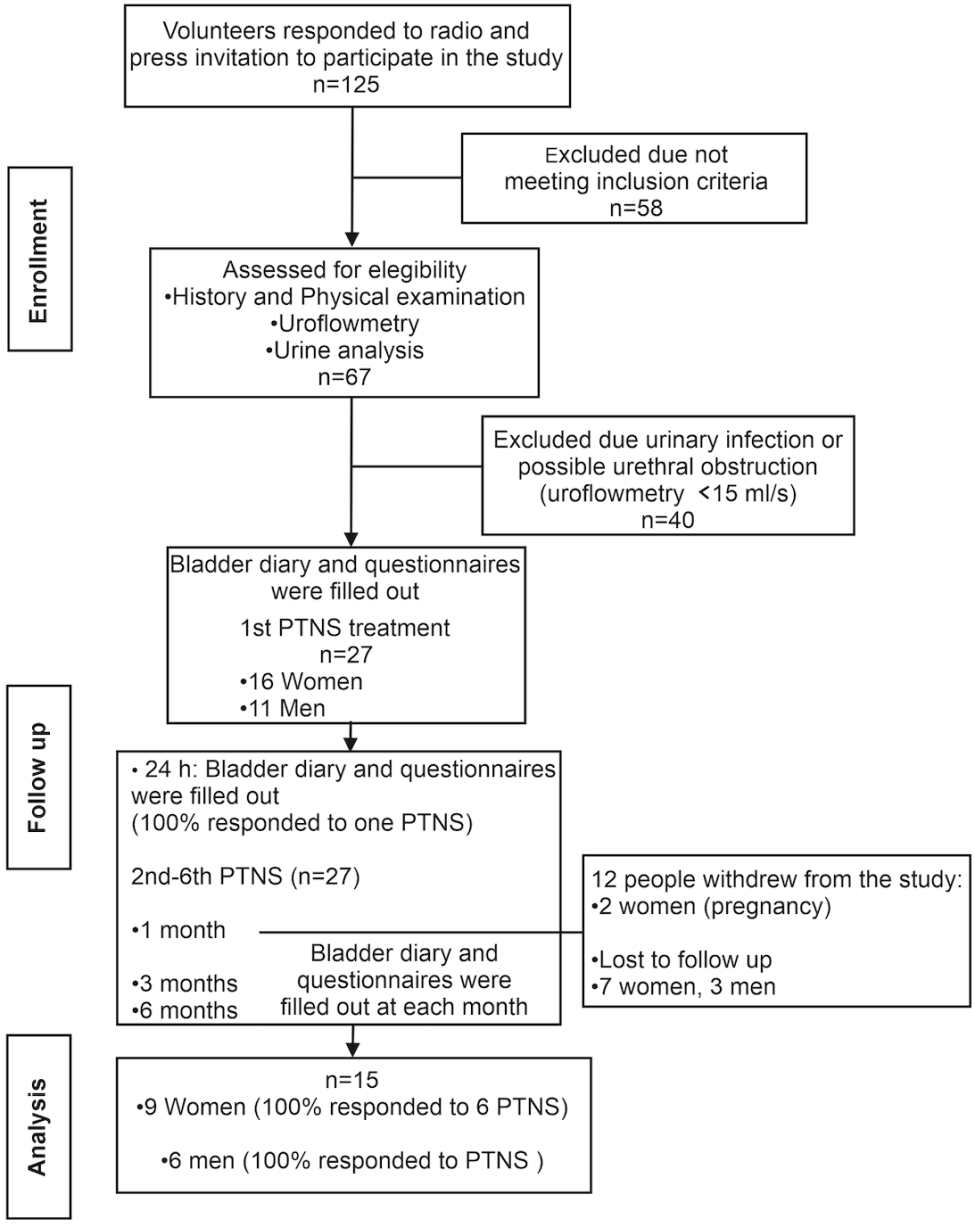
Flowchart of the patient enrollment in the study.

A total of six PTNS sessions were given to each patient over a period of 2 weeks (three PTNS sessions per week). A total of 12 volunteers opted not to continue the study after 1 month. Thus, data for PTNS analysis were obtained from 9 women and 6 men (*n* = 15) (**Figure 1**).

Patients completed a bladder diary (9, 10), the short form of the Overactive Bladder Symptom Score questionnaire (OAB-q SF Part A, symptom bother scoring) (11), and the Patient Perception of Bladder Condition (PPBC) (12) before PTNS treatment, at 24 h after the first session, and then at 1, 3, and 6 months after the six PTNS sessions (**Figure 1**).

### 2.2 Percutaneous tibial nerve stimulation protocol

Asepsis of the tibial area of the dominant foot was performed. A 34-gauge stainless steel needle was inserted 3–4 cm deep at 5 cm cephalic from the medial malleolus, just between the posterior margin of the tibia and the soleus muscle (13, 14). The electrode was connected to a low-voltage neuromuscular electrostimulator (EMS+2; Staodyn Inc., Longmont, CO, USA) for a 30-min session. The parameters for the PTNS were 20 Hz and a wave amplitude of 200 µs, with the intensity adjusted from 1 to 9 mAmp until a physiological response occurred and observed as plantar flexion of the first toe and/or fanning of the toes (15) and stopped just before pain sensation. A reference patch electrode was placed at the lateral border of the same foot. The threshold parameters determined in each person were used in the six PTNS sessions.

### 2.3 Bladder diary

A nurse assistant (same person) trained volunteers to complete a preprinted bladder diary as described elsewhere (9, 10). We included the subjects’ “time to go to sleep” and “time to wake up” to define nighttime and daytime, respectively. The nurse also provided them with a 1,000-ml graduated cup to measure the urinary volume; all volunteers were also trained on how to use the cup. The data for frequency, urgency, nocturia, and urinary incontinence were collected from the responses in the bladder diary.

### 2.4 Compensation for participation in research

All medical services, including physical examination, studies, and treatment, were free of charge as benefits of participation.

### 2.5 Statistical analysis

Data were collected in an Excel database, and statistical analysis and graphs were done using SigmaPlot software (SYSTAT, San Jose, CA, USA). The mean age and OAB evolution time were compared by sex using a *t*-test. The bladder diary parameters (i.e., frequency, urgency, nocturia, and urinary incontinence) and the OAB-q SF and PPBC questionnaire scores were analyzed using two-way repeated measures ANOVA or the Holm–Sidak method (the alpha level was set to *p* < 0.05). Clinical success was considered when the frequency decreased to ≤8 voids in 24 h and when nocturia, urgency, or incontinence decreased by ≥50%.

## 3 Results

### 3.1 Participants

No statistical differences were found for the mean age of participants (women, 48.67 ± 6.21 years; men, 56.50 ± 6.21 years; *t*-test, *p* = 0.40) or for the OAB evolution time (women, 28.44 ± 12.48 months; men, 39 ± 18.2 months; *t*-test, *p* = 0.56).

### 3.2 Efficacy of PTNS treatment on OAB symptoms

For threshold stimulation, efficacy did not vary significantly between sexes (women, 1.88 ± 0.18 mA; men, 2 ± 0.18 mA; ***t***-test, p = 0.68).

Regarding the mean voiding volume, PTNS showed a positive effect in both men and women at all time points (factor treatment: *p* = 0.055, two-way ANOVA). However, there was no significant difference when compared between sexes (*p* > 0.05). The mixed mean voiding volume (i.e., combining men and women) increased significantly after treatment (177 ± 48 vs. 208 ± 71 ml for before treatment and after 24 h and 198 ± 61, 201 ± 65, and 198 ± 63 ml at 1, 3, and 6 months, respectively; (*p* < 0.05).

**Figure 2:**
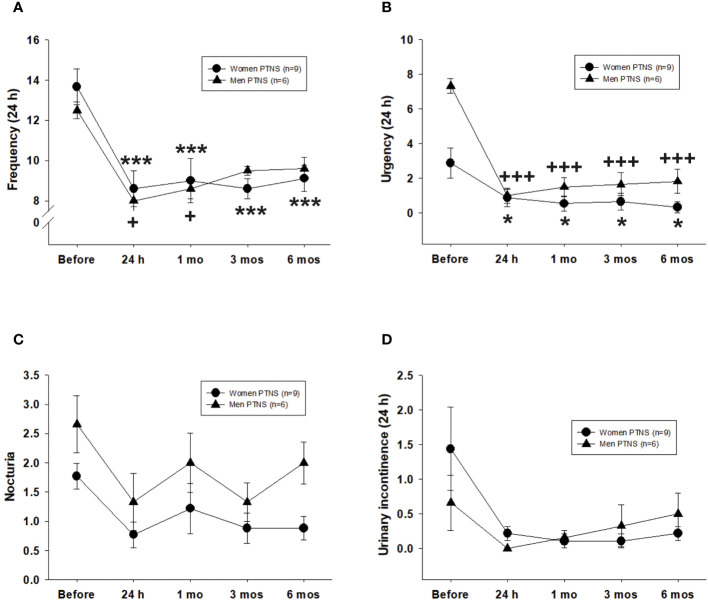
Efficacy of six percutaneous tibial nerve stimulation (PTNS) sessions on overactive bladder (OAB) symptoms, including frequency **(A)**, urgency **(B)**, nocturia **(C)**, and urinary incontinence **(D)**. Values represent the mean ± standard error of the mean. ***p < 0.001; ^+++^ *p* < 0.001 (indicating significant differences for women and men, respectively, vs. before PTNS; two-way repeated measures ANOVA).

In terms of micturition frequency, the value of this parameter before PTNS was not different between men and women (two-way repeated measures ANOVA, *p* = 0.63) (**Figure 2A**). However, sex differences were observed on the duration of the effect of treatment over time. At 24 h after the first PTNS session, the frequency of micturition decreased significantly in female patients with OAB from 13.6 to 8.6 ± 0.8 voids (range, 33%–36%; *p* < 0.01). Similar results were observed in male patients (8 ± 0.6 voids post-PTNS; range, 31%–36%).

Compared to pretreatment, the void frequency in women stayed significantly low at 1 (9.1 ± 1.1 micturition), 3 (8.6 ± 0.5 micturition), and 6 months (9.1 ± 0.6 micturition) following six PTNS sessions (two-way repeated measures ANOVA, *p* < 0.001) (**Figure 2A**). However, in men, a PTNS-induced long-lasting inhibitory effect was not observed as there were no significant differences between pretreatment *versus* 3 or 6 months after PTNS treatment (two-way repeated measures ANOVA, *p* < 0.05) (**Figure 2A**).

With regard to urgency, there were more urge episodes before PTNS treatment in men than in women (**Figure 2B**). However, no significant differences were found between sexes after PTNS treatment on either analyzed time point (two-way repeated measures ANOVA, *p* = 0.91) (**Figure 2B**). The percentage of urgency reduction ranged from 70% to 89% in women and from 75% to 88% in men. At 24 h after one PTNS session, urge episodes were significantly decreased in all women and men. Significant differences were still observed at 1, 3, and 6 months after PTNS treatment when compared to the values before treatment (two-way repeated measures ANOVA, *p* < 0.001) (**Figure 2B**).

The values for nocturia and urinary incontinence did not significantly vary between women and men, both before and after PTNS sessions (two-way repeated measures ANOVA, *p* > 0.05) (**Figures 2C, D**).

### 3.3 Clinical success by sex

According to the criteria described in Methods, clinical success was reached for most bladder diary parameters at 24 h after the first PTNS session. Independent of the sex, the positive effect of PTNS on most OAB symptoms persisted 6 months after the treatment.

**Figure 3:**
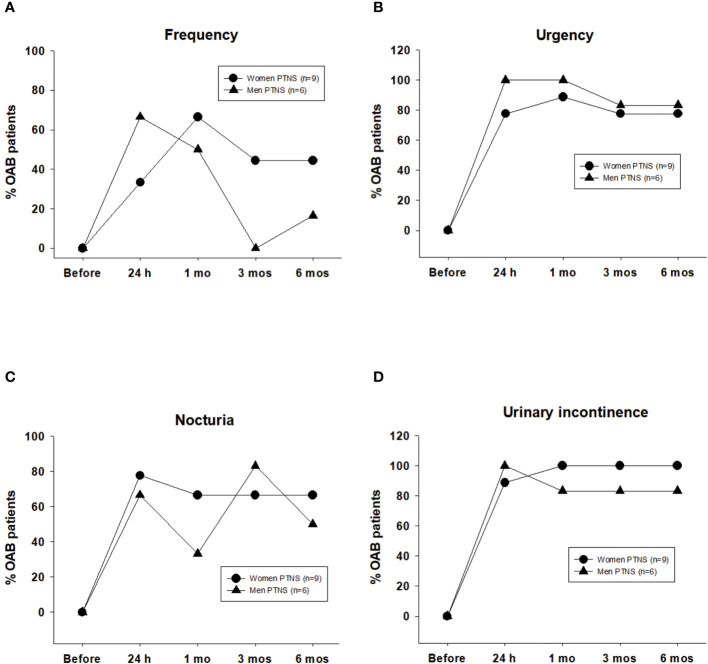
Clinical success induced by six percutaneous tibial nerve stimulation (PTNS) sessions determined when frequency decreased by ≤8 voids in 24 h **(A)** and once the symptoms of urgency **(B)**, nocturia **(C)**, and urinary incontinence **(D)** decreased by ≥50%. Values represent the percentage of patients with clinical success.

With regard to frequency, after 24 h of a single PTNS session, 33% of female patients with OAB showed clinical success. After six PTNS sessions, the percentages of patients with clinical success were 66% at 1 month and ~40% after 3 and 6 months. In men, high values of clinical success (>60%) were observed only after 24 h of the first PTNS session and after 1 month of six PTNS treatments (~50%) (**Figure 3A**).

After PTNS treatment, the clinical success for urgency and urinary incontinence was high, between 77% and 100% in both sexes (**Figures 3B, D**).

Clinical success in nocturia was higher than 60% in both sexes, with the exception at 1 month in men (33%) (**Figure 3C**).

### 3.4 OABq-SF and PPBC scores

**Figure 4:**
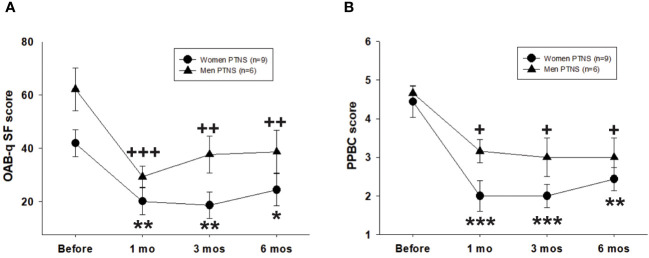
Efficacy of six percutaneous tibial nerve stimulation (PTNS) sessions on the short form of the Overactive Bladder Symptom Score questionnaire (OAB-q SF) **(A)** and the Patient Perception of Bladder Condition (PPBC) **(B)**. Values represent the mean ± standard error of the mean. *p < 0.05, **p < 0.01, ***p < 0.001; ^+^ *p* < 0.05, ^++^ *p* < 0.01, ^+++^ *p* < 0.001 (indicating significant differences for women and men, respectively, vs. before PTNS; two-way repeated measures ANOVA).

The OABq-SF score was significantly decreased in 100% of OAB patients. Compared to those before PTNS (women, 41.8 ± 5.6; men, 62.1 ± 8.4), the OABq-SF scores were significantly reduced at 1, 3, and 6 months after treatment in both sexes (two-way repeated measures ANOVA, *p* < 0.001) (**Figure 4A**).

The PPBC score was significantly decreased after treatment in both sexes and at all time points of the study (two-way repeated measures ANOVA, *p* < 0.001) (**Figure 4B**). The effect of PTNS was more pronounced in women (*p* < 0.001) than in men (*p* < 0.05), although there were no significant differences between sexes (two-way repeated measures ANOVA, *p* > 0.05).

## 4 Discussion

PTNS is an alternative and minimally invasive treatment for OAB, a burden that affects the health and economy of many people worldwide (2), accounting in the US for about 69.95% of the direct total healthcare budget (16). Currently, a 10- to 12-session PTNS protocol is mostly employed (7). Only two studies have evaluated the efficacy of a decreased number of sessions in obtaining clinical benefits on OAB symptoms in women (17, 18).

To our knowledge, the present study is the first report demonstrating clinical improvements of OAB symptoms and a positive enhancement on the quality of life beginning from the first session of PTNS in both sexes.

The sustained clinical impact of PTNS over a 6-month period is particularly noteworthy, most probably resulting in a significant reduction in the temporal and economic burden associated with patient treatment. Consequently, the present study strongly suggests changing the paradigm of reducing 12 PTNS sessions to only six sessions and further tailoring it for restimulation according to the requirements of each patient. Therefore, it is imperative to redefine the approach of treating OAB using PTNS, especially now that implanting of the tibial nerve stimulators has become available (19). In addition, our investigation has undertaken a relatively underexplored domain, the sex-based effects of PTNS. In this sense, our results unveiled novel insights into the sex-specific variations in the enduring impact of PTNS. We found no differences between the sexes regarding the effect of PTNS on most of the bladder diary parameters. However, we discovered that women experienced prolonged clinical success in terms of reduced urinary frequency and an enhanced quality of life, while men exhibited superior outcomes regarding urgency reduction.

In agreement with our findings, Yoong et al. (2010) reported in women a positive effect of 6-week sessions (one session per week) in reducing the symptoms of OAB. They reported a median duration of positive response of 3 weeks, after which bothersome symptoms started to reappear (17). However, in our study, the positive effects of treatment with six PTNS sessions on the diary parameters and the relief of OAB bothersome symptoms were long-lasting, up to 6 months in women. Considering that both studies applied a protocol of six PTNS sessions, the difference could be related to the interstimulation interval: they applied one PTNS per week, while we did three PTNS sessions per week. Whether the frequency of stimulation influences the effect of PTNS is unknown. Further studies are required.

In another study, it was reported that the success rates for OAB symptoms were higher in women who underwent 12 weekly PTNS sessions than in those who underwent 8 sessions. Differences were found in the Patient Global Improvement Questionnaire, but not in the OABq-SF. However, the study was not controlled, and some of the patients were also taking oral medications, using vaginal estrogen, presented with urinary tract infection, or had a neurological diagnosis. In addition, the authors recognized that the between-group differences could be related to the definition used for success. Therefore, they concluded that reducing the number of PTNS sessions is recommended (18).

It is important to note that, in our study, clinical success was defined as a decrease in urinary frequency to ≤8 voids in 24 h, as previously reported (20). However, a review described the reduction of micturition frequency by PTNS as having a limit of 3.67 events (7). In support of these authors, we suggest that clinical success must be redefined as a reduction of urinary frequency of ≤4 voids in 24 h.

Our finding of the clinical effect on OAB symptoms of a single PTNS at 24 h after stimulation suggests that plastic changes can occur in the neural circuitry controlling the lower urinary tract (LUT) immediately after the first PTNS session. In fact, it has been demonstrated that PTNS induced changes at the cortical level, with long-lasting neuroplastic reorganization enduring for at least 24 h after neuromodulation (8).

Based on clinical and basic research, it has been postulated that bladder neuromodulation may occur by inhibiting the bladder’s sensory and motor pathways at different levels of the nervous system (8, 21, 22). At the peripheral levels, PTNS could activate somatic afferent nerves that stimulate spinal inhibitory interneurons located at the L5–S3 spinal segments. Inhibition of bladder afferent signaling and bladder preganglionic neurons (21) may be achieved through the activation of the μ, κ, and δ opioid receptors (22), resulting in improvement of bladder compliance and reduced overactivity.

Although we do not know the duration of the neuromodulation, considering that the effect of six PTNS sessions persisted for up to 6 months, it is suggested that the neuroplastic changes in central pathways controlling bladder function are long-lasting. It has been reported that the 12 PTNS protocol plus a tailored intermittent PTNS stimulation could maintain the positive effects on OAB for up to 2 years (23), or even 3 years (24).

Regarding sex differences, we found that PTNS treatment significantly reduced the bladder diary parameters in both men and women. This finding indicates that neuromodulation occurs independently of the sex and that PTNS may be used to treat OAB regardless of sex. Similarly, the effectiveness of sacral neuromodulation in reducing the symptoms of OAB was comparable between men and women (25).

A similarity in the efficacy of PTNS on OAB between sexes was not expected as the intensity of the symptoms is usually higher in women than men. In accordance with our results, sex-specific differences in relation to individual OAB symptoms (3) and the underlying causal factors have been reported. In contrast to women, the LUT symptoms in men have mainly been related to bladder outlet obstruction due to benign prostatic hyperplasia (BPH), which is more prevalent with age (26) and can contribute to increased urinary frequency and urgency. In women, the mechanisms underlying increased urinary frequency may be neurological due to nerve and muscle damage that can occur in the urogenital apparatus during pregnancy and child delivery (27). The similar clinical efficacy between sexes in our results could be due to the fact that our study excluded bladder outlet obstruction as a causal factor (prostate enlargement and low urine flow rate were considered as part of the exclusion criteria).

We cannot exclude the possibility that the efficacy of PTNS may vary between sexes under other conditions. In fact, we found sex-dependent differences regarding the duration of the effects of PTNS. For example, clinical success for improving urinary frequency was sustained for up to 6 months in women, but not in men. This gender disparity could suggest several hypotheses, including the possibility that the underlying causes of high urinary frequency in male patients may not be solely mechanical in nature. Unfortunately, studies including sex as an independent variable in evaluating the clinical success of PTNS are scarce. In addition, the limited studies including men in their research only had a few male participants (7). However, there is an overall OBA prevalence in men and women, which increases with aging in both sexes. Thus, OAB treatment in men is a topic that deserves increased attention.

### 4.1 Study limitations

The present study has some limitations. Firstly, we did not include a placebo group for PTNS stimulation based in part to the fact that other studies have demonstrated that sham PTNS stimulation did not change the OAB symptoms (14, 28). Secondly, some patients discontinued their participation in the study at different stages of the treatment, a situation that has been described in other studies (22, 23, 29, 30). It is not known whether it was due to substantial OAB improvement in the patient or due to other factors such as traveling a long distance to access clinical service or even a financial burden for attending appointments during a 6-month period. Thirdly, although there were few male patients included in the study, the results and the effects of PTNS were promising for the implementation of additional studies in men. Finally, the follow-up outcomes were a symptom-based assessment only; however, the use of a bladder diary and the OAB-q SF and PPBC questionnaires has been validated as reliable outcomes and widely utilized in several OAB studies (9–12). Furthermore, in our study, the enrollment criteria for patients with OAB included the performance of a clinical history, a physical examination, a uroflowmetry assessment, and a bladder diary, which further validated the eligibility of OAB for enrollment. We were not able to include urodynamic studies due to the homogeneous nature of the improvements seen in patients and because of the economic costs associated with a cystometric evaluation. We recognize that additional basic and clinical studies are necessary to further endorse our findings in order to understand the physiological mechanisms of PTNS treatment and the stimulation variables to improve both the efficacy and cost reduction during OAB treatment.

## 5 Conclusions

A shortened protocol of six PTNS sessions significantly improves the symptoms of OAB and the quality of life, with long-lasting effects in both men and women. Sex differences slightly affect the mean duration of treatment in terms of frequency and perception of improvement in quality of life. The long-lasting effects of PTNS strongly suggest the potential implementation of fewer PTNS sessions to effectively alleviate the symptoms of OAB, which would reduce the time and economic costs of treatment.

## Data availability statement

The raw data supporting the conclusions of this article will be made available by the authors, without undue reservation.

## Ethics statement

The studies involving humans were approved by Public Health Ethics Committee, Protocol Public Registry Number ISRCTN15733799. The studies were conducted in accordance with the local legislation and institutional requirements. The participants provided their written informed consent to participate in this study.

## Author contributions

CP-M: Investigation, Methodology, Project administration, Supervision, Writing – original draft. JP-G: Data curation, Formal analysis, Writing – review & editing. IBV-D: Writing – review & editing, Project administration, Resources, Supervision. AM: Writing – review & editing, Formal analysis. YC: Conceptualization, Investigation, Methodology, Supervision, Writing – review & editing.
